# Unlocking gut microbiota potential of dairy cows in varied environmental conditions using shotgun metagenomic approach

**DOI:** 10.1186/s12866-023-03101-7

**Published:** 2023-11-16

**Authors:** Faheem Ahmed Khan, Nuruliarizki Shinta Pandupuspitasari, Chunjie Huang, Windu Negara, Bilal Ahmed, Ezi Masdia Putri, Puji Lestari, Tri Puji Priyatno, Ari Prima, Vita Restitrisnani, Maman Surachman, Sindu Akhadiarto, I Wayan Angga Darmawan, Dimar Sari Wahyuni, Herdis Herdis

**Affiliations:** 1 Research Center for Animal Husbandry, National Research and Innovation Agency, Jakarta Pusat, 10340 Indonesia; 2 Department of Zoology, Faculty of Science and Technology, University of Central Punjab, Lahore, 54782 Pakistan; 3 Laboratory of Animal Nutrition and Feed Science, Animal Science Department, Faculty of Animal and Agricultural Sciences, Universitas Diponegoro, Semarang, Indonesia; 4 Department of Biological Engineering, Massachusetts Institute of Technology, Massachusetts, Cambridge 02139 USA; 5 PT Bumi Yasa Svarga, Sukabumi, 43152 Indonesia; 6 Institute of Reproductive Medicine, School of Medicine, Nantong University, Nantong, 226001 China; 7 Research Organization of Agriculture and Food National Research and Innovation Agency, Bogor, Indonesia

**Keywords:** Gut microbiota, Dairy cows, Shotgun, Food security, Microbes

## Abstract

Food security and environmental pollution are major concerns for the expanding world population, where farm animals are the largest source of dietary proteins and are responsible for producing anthropogenic gases, including methane, especially by cows. We sampled the fecal microbiomes of cows from varying environmental regions of Pakistan to determine the better-performing microbiomes for higher yields and lower methane emissions by applying the shotgun metagenomic approach. We selected managed dairy farms in the Chakwal, Salt Range, and Patoki regions of Pakistan, and also incorporated animals from local farmers. Milk yield and milk fat, and protein contents were measured and correlated with microbiome diversity and function. The average milk protein content from the Salt Range farms was 2.68%, with an average peak milk yield of 45 litters/head/day, compared to 3.68% in Patoki farms with an average peak milk yield of 18 litters/head/day. Salt-range dairy cows prefer S-adenosyl-L-methionine (SAMe) to S-adenosyl-L-homocysteine (SAH) conversion reactions and are responsible for low milk protein content. It is linked to *Bacteroides fragilles* which account for 10% of the total *Bacteroides*, compared to 3% in the Patoki region. The solid Non-Fat in the salt range was 8.29%, whereas that in patoki was 6.34%. Moreover, *Lactobacillus plantarum* high abundance in Salt Range provided propionate as alternate sink to [H], and overcoming a *Methanobrevibacter ruminantium* high methane emissions in the Salt Range. Furthermore, our results identified ruminant fecal microbiomes that can be used as fecal microbiota transplants (FMT) to high-methane emitters and low-performing herds to increase farm output and reduce the environmental damage caused by anthropogenic gases emitted by dairy cows.

## Introduction

Global warming is challenging the food security of an expanding population by impacting gut microbiota diversity and its ecological interactions, especially for commensals, predators, and symbionts. Such changes in the microbiome pose an immediate threat and cause host system dysfunction [1]. The gut microbiota has been implicated in several biological processes across different species, and its role in dairy cow milk production and quality is of interest to the dairy industry and the well-being of the rural economy [2]. Microbial products help in food fermentation, methane and nitrogen emissions, and fiber breakdown [3]. Bifidobacterium, Ruminococcus, and Fibrobacter help in cellulose digestion, whereas Succinovibrinonaceae impacts methane emissions [4–6]. Volatile fatty acids are compounds formed by microbial fermentation in the gut of ruminants that provide an energy source to the host [7, 8]. For example, *Clostridium sticklandii* is involved in NH_3_ production, whereas *Rosebura* species produce butyrate [9–11].

To explore the gut microbiota, feces remain the most important source [12], and present-day molecular biology techniques and next-generation sequencing (NGS) can help identify complex microbial communities. Fecal microbial composition depends mainly on diet, environment, and health status [13, 14]. Kim and Wells’s meta-analysis report showed 10 phyla, 17 classes, 28 orders, 59 families, and 110 genera present in the fecal microbiome of cows, of which the most common phyla reported in several studies were Firmicutes. Proteobacteria and Bacteroidetes [15].

Animal microbiota composition depends on various factors, including the environment, which influences animal feed intake, and host factors, such as immunity and metabolism, resulting in diversified microbiomes [16]. Different feed components influence various classes of bacterial and archaeal groups, such as methanobacteria, fiber, and fat digesters [17]. Dairy cows depend on microbes to obtain various products for the digestion of proteins, fibers, vitamins, and minerals from the diet [18], and the balance of these components is crucial for the overall output with regard to the quality and quantity of milk [17]. Besides diet, environmental factors such as water quality, temperature, altitude, latitude, and flora influence the host gut microbiota, causing a multitude of phenotypes [19–21].

In the present study, we evaluated the diversity, abundance, and function of gut microbiota from dairy farms in ecologically diverse zones of Pakistan, namely Chakwal, an arid hilly zone recognized for its great fauna diversity, Salt Range with high availability of pink salt, Patoki as lowland, and a polluted area with the inclusion of animals from local farmers fed concentrate diets and grassland. The objective was to determine microbiomes for better productivity and lower methane emissions and to recommend microbiomes with better performance for use as fecal microbiota transplant (FMT) to low-performing animal herds in original environmental conditions. In this regard, we thoroughly studied and compared the metabolic pathways among the groups to evaluate and logically explain the mechanisms behind each phenotype. The results identified potential fecal microbiomes for use as FMT in compromised herds.

## Materials and methods

### Animal farms selection and metagenomic analysis

Dairy cow farms from diverse regions of Pakistan, that is, Chakwal, Patoki, Salt range, and dairy cows of local farmers, were selected for the study. Fecal samples from healthy cows (five cows were selected for each zone) were collected pooled and homogenized, and DNA was extracted using a QIAMP Fast DNA Mini Stool Extraction Kit. The quality of the extracted DNA was then tested. Sample quantification was performed using a Qubit fluorometer, and its integrity was checked by agarose gel electrophoresis. For genomic DNA, 1% agarose gel was run for 40 min at 100 V, whereas the PCR product was run for 50 min on a 2% agarose gel at 80 V.

After DNA quality checks, a genomic DNA library was constructed by end repair, adding A to the tail, and performing PCR amplification. The Qubit 2.0 was used to quantify libraries, which were then diluted to 2 ng/ul to check for the insert size using Agilent 2100. Library quality was maintained using qPCR. Libraries that passed quality checks were sequenced using a Novaseq 6000. Sequenced data were assembled to generate scaffolds. Scaft gene prediction was performed using MetaGeneMark. All unique genes were then used to create a gene catalog, and the genes in different samples were calculated using the gene catalog and the clean data. Annotation information for the gene catalog was obtained by performing BLAST against the micro NR database. The functional annotation was achieved by performing Blast searches against KEGG, eggNOG, and CaZY databases.

### Fecal sampling

Fecal samples were collected from selected dairy animals soon after defecation. Samples of dairy cow stool were collected in RNA/DNA shield tubes. After collection in tubes and proper labeling, a tag id was written on the tubes. Fecal samples from each region were pooled separately, homogenized, and DNA was extracted for shotgun metagenomic sequencing.

### Milk sampling

Milk samples from dairy cows of the selected farms were collected and tested for fat and protein content. Milk samples were stored without preservatives for downstream processing. Milk samples from the same animals were analyzed for their qualitative traits to associate the data with the shotgun metagenomic data.

## Results and discussion

### Data preprocessing

DNA was isolated from fecal samples of dairy cows from farms in Chakwal, Salt Range, Patoki and from samples of local farmers’ dairy cattle for constructing metagenomic libraries. The raw sequencing reads obtained were 6.88 Gb, 6.15 Gb, 6.70 Gb, and 7.37 Gb for the Chakwal Patoki, local, and saline ranges, respectively. The clean data obtained was 6.86 Gb, 6.14 Gb, 6.68 Gb, and 7.34 Gb, respectively. Q_30 clean data corresponded to a sequencing error rate of less than 0.001 in the Chakwal study. The Patoki, local, and salt ages were 90.40%, 89.60%, 90.43%, and 90.35%, respectively. The NovaSeq 6000 platform was used for sequencing. All scaftigs were counted in the assembled results with the contribution of scaftigs to each sample. Unutilized reads were subjected to mixed assembly, maintaining the same assembly parameters. The data preprocessing statistics are listed in Table 1.

**Table 1:** Statistics of data preprocessing representing samples, the fragment length used for library construction, Raw Data size of each sample, clean data obtained after quality check, Clean Q_20 data percentage with an error rate less than 0.01, Clean Q_30 with an error rate less than 0.001. The GC content and ratio of clean data over raw data

Sample	Insert size	Raw Data (G)	Clean Data (G)	Clean_ Q20%	Clean_Q30 (%)	Clean GC (%)	Effective
Chakwal	350	6.88	6.86	96.53	90.40	42.41	99.723
Patoki	350	6.15	6.14	96.36	89.60	41.33	99.718
Local	350	6.70	6.68	96.53	90.43	43.09	99.787
Salt_Range	350	7.37	7.34	96.50	90.35	43.87	99.612

### Metagenome assembly

**Fig. 1:**
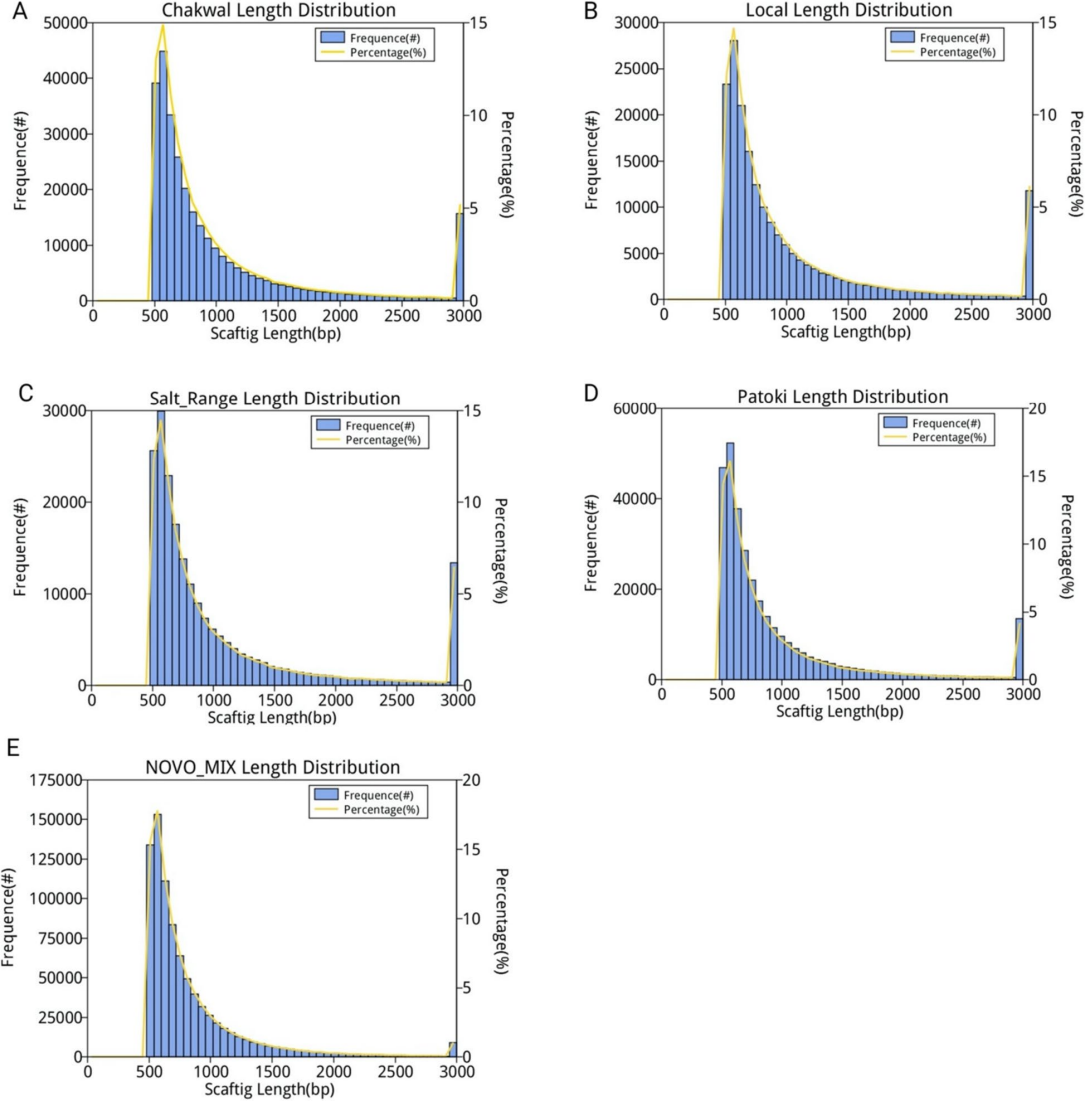
Distribution of scaftigs. Certain lengths of scaftigs are given at Y1-axis named “Frequence; Certain length of Scaftigs; Percentage of scaftigs to total scaftigs is given at Y2-axis named “Percentage (%)”; The X-axis titled “Scaftigs Length (bp)” indicates the length of Scaftigs. **A** Chakwal Length distribution, **B** Local length distribution, **C** Salt Range length distribution, **D** Patoki Length Distribution, **E** NOVO-MIX length distribution

The clean data were assembled using Soapdenovo. The longest N50 was considered the final result. The scaffolds were interrupted at N to obtain the scaffolds. Clean data were aligned to scaftigs using SoapAligner. Subsequently, a mixed-assembly analysis was conducted by combining all the unutilized reads. Scaftigs of less than 500 bp were filtered, and the remaining effective scaftigs were used for further analysis. The scafting statistics are presented in Table 2. The distribution of scaffolds in the samples is shown in Fig. 1.

**Table 2:** Statistics of scaftigs. The total length of all the scaftigs, number of scaftigs, average scaftigs length, N50 or N90 represent the length at 50% or 90% of the total length of scaftigs and the maximum length of the scaftigs is provided for each sample

SampleID	Total length (bp)	Number of Scaftigs	Average length (bp)	N50 Length (bp)	N90 Length (bp)	Max length (bp)
Chakwal	374,089,007	300,980	1,242.90	1,404	578	386,952
Patoki	356,611,352	324,047	1,100.49	1,154	561	168,916
Salt_Range	287,013,105	206,419	1,390.44	1,778	593	671,517
Local	256,340,267	190,855	1,343.12	1,648	589	251,252
NOVO_MIX	732,189,530	861,623	849.78	830	542	30,435

### Gene prediction and abundance analysis

**Fig. 2:**
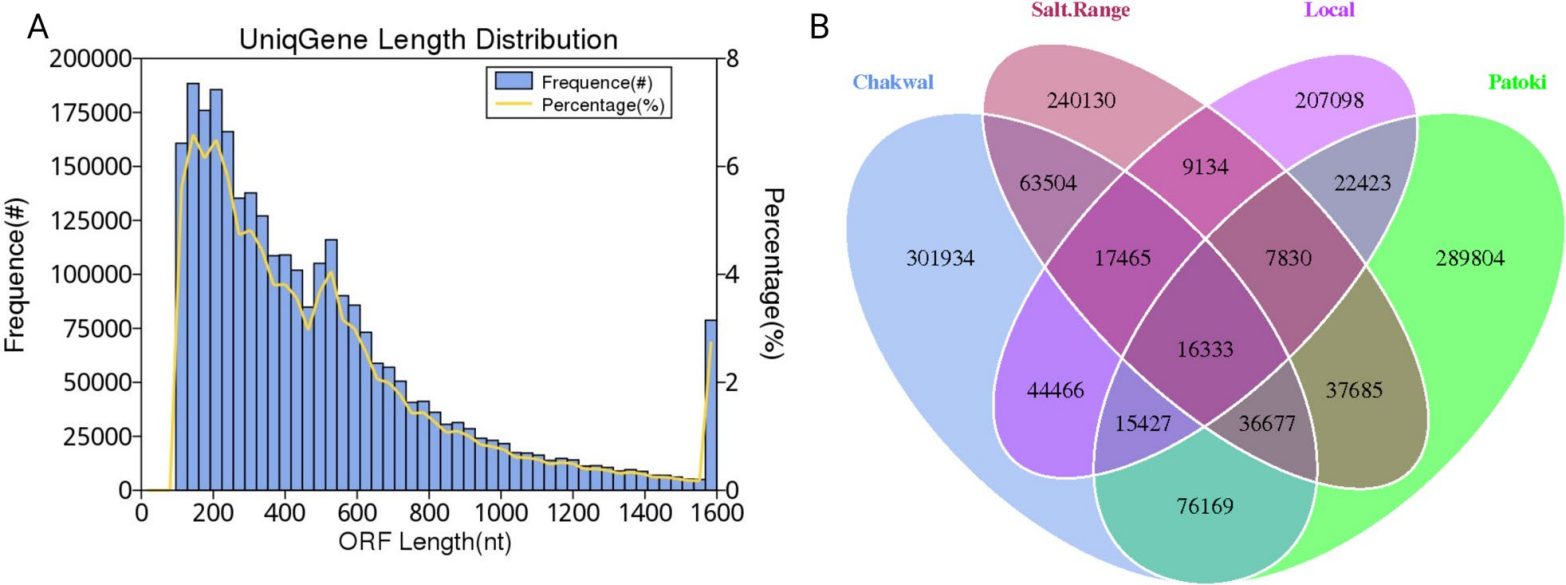
Gene prediction and abundance analysis. **a** Gene Distribution catalog. The number of genes plotted against the Y1-axis, genes percentage against Y2-axis, and genes length against the X-axis. **b** Venn Diagram to show gene numbers in samples. The number of common genes between/among samples is shown by overlap; the other parts represent the number of special genes in samples

The open reading frame (ORF) was predicted using MetaGeneMark utilizing scaftigs greater than or equal to 500 bp. CD-HIT was used to dereplicate the ORF results to generate a gene catalog in which the longest ORF was chosen as the representative gene, that is, the unigene. Furthermore, clean data were mapped to the gene catalog using SoapAlighner to calculate the mapping reads. The gene catalog statistics are given in Table 3, and the distribution is shown in Fig. 2a. The common and peculiar genes in each sample are shown in Fig. 2b.

**Table 3:** Statistics of gene catalog. The number of genes are given as ORFs NO. Genes containing only stop codon is represented as integrity: end. Genes having both start and stop codon is represented as integrity: all. Genes without start and stop codons is shown as integrity: none. The total length of gene catalog is given as (Mbp) million. Average length in the Gene catalog is shown as the average length. GC content of the gene catalog is given as GC Percent

ORFs NO	2,858,457
Integrity:end	753,041 (26.34%)
Integrity:all	1,302,628 (45.57%)
Integrity:none	170,302 (5.96%)
Integrity:start	632,486 (22.13%)
Total Length (Mbp)	1,455.04
Average Length (bp)	509.03
GC percent	44.3

### Relative abundance of bacteria in managed farms of Chakwal, Salt range, Patoki, and dairy cattle of local farmers

**Fig. 3:**
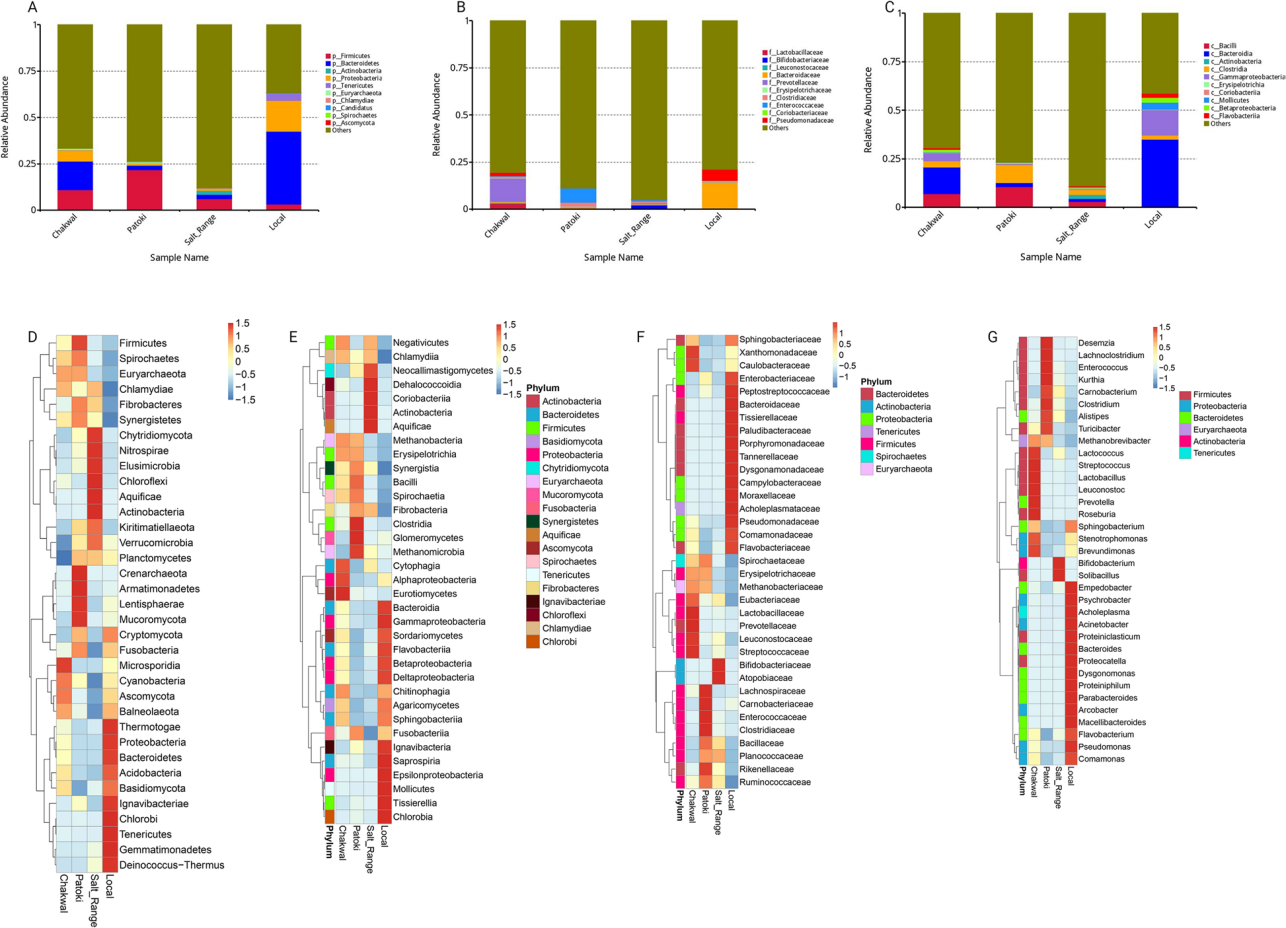
The relative abundance of fecal gut microbiota. **A** Phylum Level, **B** Class level, **C** family level **D** Heatmap of top 35 phyla **E** Heatmap of top 35 class members **F** Heatmap of top 35 families **G** Heatmap of top 35 genera

A shotgun metagenomic approach identified significant differences in bacteria at the phylum level. The phylum Firmicutes constituted 81% of the total bacteria in the Patoki region, followed by 49% in the Salt Range, 33% in Chakwal, and only 6% in the dairy cattle of local farmers. Firmicutes play a significant role in feed conversion, lower methane emissions, and higher milk yields by the production of short chain fatty acid (SCFA) [22]; however, high abundance is associated with the overproduction of volatile fatty acids (VFA), which results in ruminant metabolic disorders, including subacute ruminal acidosis, leading to a significant reduction in milk yield [23]. It became evident from our results that significantly higher milk yields were recorded in the Salt Range region, that is 45 L/day, compared to 18 L/day in Patoki. It is pertinent to note that milk yields in the Chakwal region were 28 L/day. The class Bacilli bacteria present in ruminants are known for their involvement in several metabolic pathways, including breakdown of cellulose and hemicellulose, production of VFAs as an energy source, and overall improved health and increased milk yield if present in appropriate abundance; however, its high abundance is associated with the production of harmful metabolites, including lactic acid, which is a major cause of acidosis where the pH of the rumen becomes too acidic and results in reduced milk yield, poor feed intake, and other health issues [15]. It was observed that the bacterial class Bacilli was highest in the Patoki region and comprised 32% of total bacteria as compared to 26% in the Salt Range, which shows another dysbiosis of the bacterial class that is responsible for lower milk yields in Patoki. To establish an appropriate abundance of bacilli, a well-balanced diet with less easily fermentable carbohydrates is provided to dairy cows to tackle the imbalance of metabolites. The bacterial class Bacteroidia can maintain a healthy microbiome, resist acidosis by preventing the overgrowth of harmful bacteria, and was also found to be at the lowest in the Patoki region (9%). Bacteroidia is also associated with the production of beneficial metabolites, including SCFA, to help accomplish the energy needs of dairy cows and was the highest in dairy cows of local farmers (48%), suggesting a reasonably high yield of 22 litters/day. The relative abundances of the bacterial phyla, classes, and families are provided in Fig. 3A, B, and C, respectively. Heatmaps of the top 35 relative genera and species are shown in Fig. 3D, E, F, and G.

*Lactobacillus plantarum* is a species of lactic acid bacteria [24] commonly found in the gastrointestinal tract of animals, including dairy cows. In recent years, there has been growing interest in using *L. plantarum* as a probiotic in dairy cow nutrition because of its potential health benefits for both the cow and the milk produced, and its role in protecting against mastitis [25]. Some reported benefits of *L. plantarum* supplementation in dairy cows include improved rumen fermentation and digestion, increased milk production, reduced incidence of mastitis and other infections, and improved milk quality [25, 26]. *L. plantarum* is believed to achieve these benefits by promoting the growth of beneficial bacteria in the gut, enhancing immune function in cows, and reducing inflammation [27]. It was observed that the abundance of *L. plantarum* was 16% of that of Lactobacillus in Salt Range, compared to 3% in Patoki, 0.5% in Chakwal, and absence in the dairy cows of local farmers. As reported, *L. plantarum* has a positive impact on milk yield and quality has been observed in dairy cows of the Salt Range, with an average milk yield of 45 L per head per day. It was also observed that *Lactobacillus acidophilus* abundance was the highest in Salt Range dairy cows, which improved milk quality by reducing somatic cell count and providing resistance against mastitis. It has been reported that *L. plantarum* plays an important role in increasing beneficial bacteria including *L. acidophilus* to help improve host health synergistically [28].

### Functional annotation of dairy cows in managed farms of Chakwal, Salt range, Patoki and dairy cattle of local farmers

**Fig. 4:**
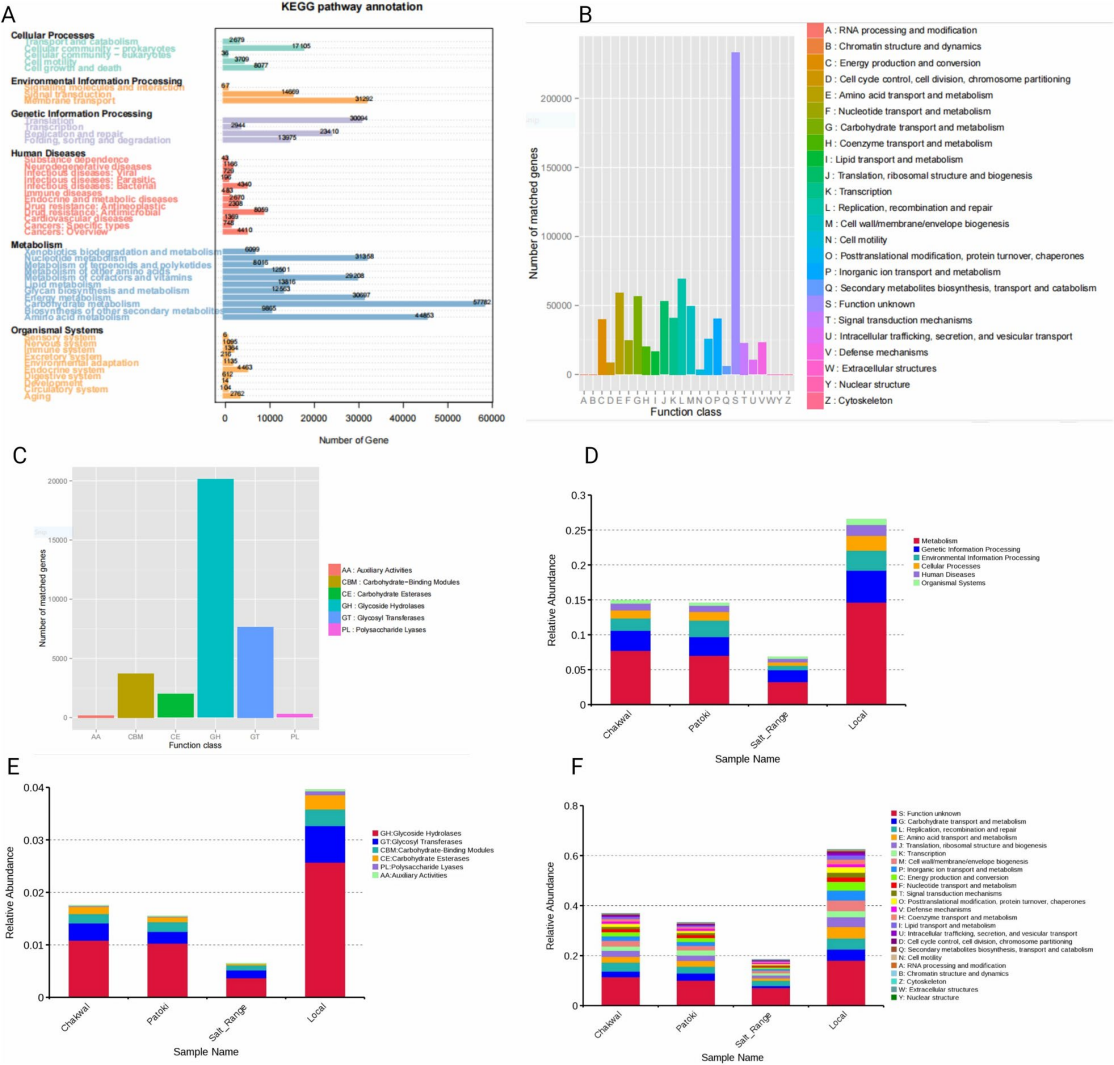
Functional annotation of fecal gut microbiota in dairy cows. **A** KEGG database annotations. **B** eggNOG database annotation. **C** CAZy database annotation. **D** KEGG Relative abundance of function. **E** CAZy Relative abundance. **F** eggNOG Relative abundance

Community physiology can be clarified using the collective functions that are encoded in the genomes of organisms that live together in a community, and can be achieved by employing shotgun metagenomic sequencing. Protein-coding sequences were mapped against KEGG [29], egg NOG [30], and CAZy [31]. Gene numbers and relative abundances were calculated at each level. UniGene annotation results for each database are shown in Fig. 4A, B and C. It can be seen in Fig. 4A that UniGene annotation to the KEGG database is the highest in metabolism and specifically in carbohydrate metabolism [57782] followed by amino acid metabolism [44853], environmental processing where membrane transport genes annotated are 31,292, genetic information processing where UniGene corresponds to translation was 30,094 followed by replication and repair, that is, 23,410, and cellular processes where Unigene annotation to cellular community – prokaryotes is 17105.

The UniGene annotated to the eggNOG database shown in Fig. 4B revealed that the highest number of genes were annotated to replication, recombination, and repair, followed by amino acid transport and metabolism, and carbohydrate metabolism and transport. A significant number of genes were annotated for inorganic ion transport and metabolism, posttranslational modification, protein turnover, and chaperones. UniGene annotated to the CAZy database had the highest number of genes annotated to glycoside hydrolases, glycosyltransferases, and carbohydrate-binding modules.

The relative abundance of genes for metabolism is highest in dairy cows from local farmers with the lowest in salt range, as shown in Fig. 4D, while the relative abundance of genes in CAZy shows the highest number of glycoside hydrolases in dairy cows from local farmers and lowest in salt range and its corresponding abundance is also shown in Fig. 4E, when annotated to eggNOG database where the relative abundance of genes for metabolism was highest in dairy cows from local farmers followed by Patoki and Chakwal shown in Fig. 4F.

### Gut microbiota affects metabolic pathways to affect milk yield, milk fat, and milk protein contents

mPATH analysis is used to analyze microbiome data, which involves analyzing the gene expression profiles of microbial communities under different environmental conditions or disease states [32]. In microbiome studies, mPATH analysis can identify differentially regulated microbial pathways associated with specific environmental conditions or disease states [5]. To perform mPATH analysis of the microbiome data, gene expression profiles of the microbial communities were first generated using techniques such as metagenomics. These gene expression profiles were then used to identify differentially expressed microbial genes under different environmental conditions or disease states. Next, the differentially expressed microbial genes were mapped to microbial pathways using pathway databases such as KEGG. The analysis shows shared and unique pathway information among the compared samples, where nodes represent chemicals and lines represent reactions. Red corresponds to shared reactions, blue corresponds to unique reactions in sample A, and green represents unique reactions in sample B.

**Fig. 5:**
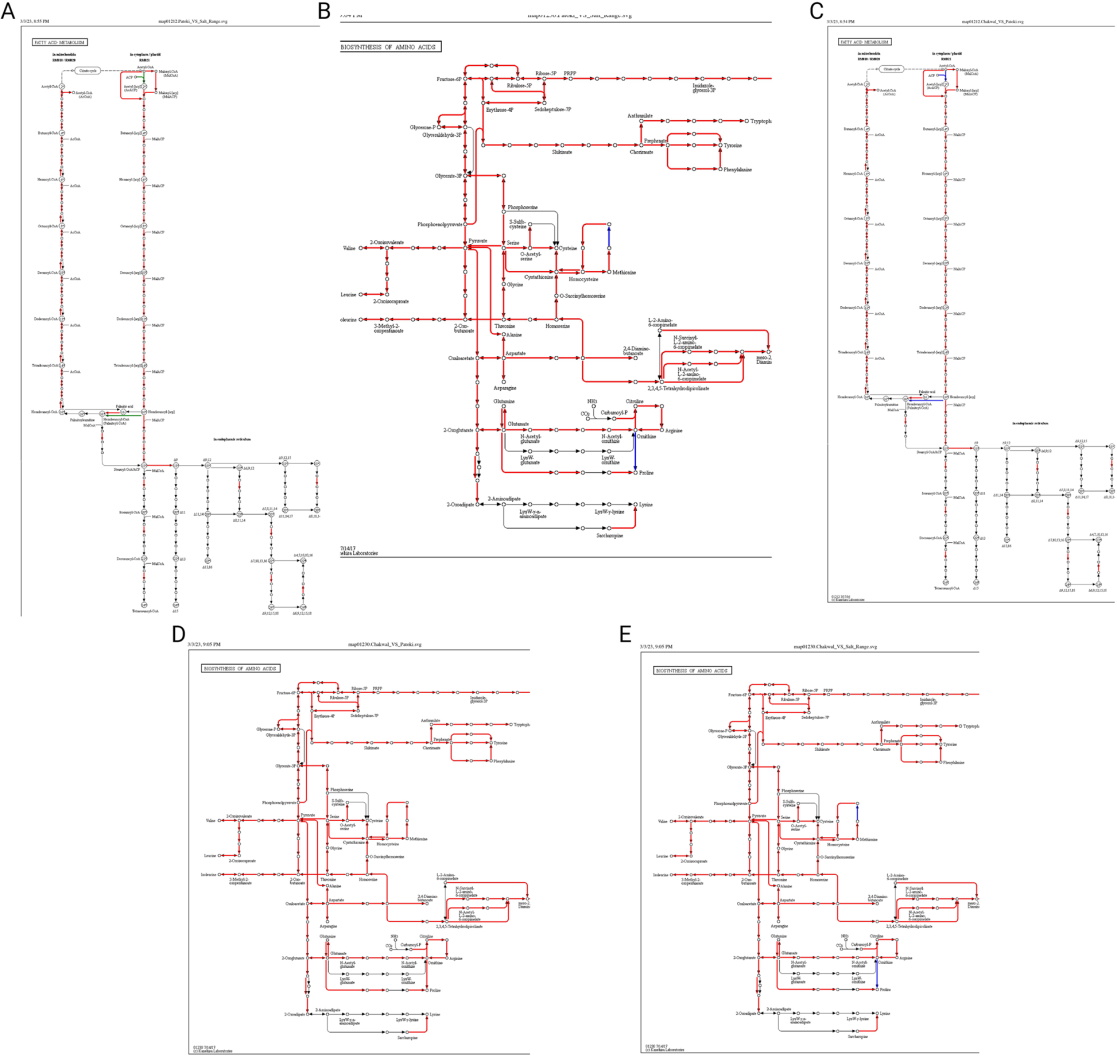
Comparison of Fatty acid metabolism and Amino acid Biosynthesis among different samples. **A** Fatty acid Metabolism in Patoki vs Salt Range, **B** Amino acid Biosynthesis comparison between Patoki vs Salt Range, **C** Fatty Acid Metabolism Chakwal vs Patoki, **D** Amino acid Biosynthesis comparison Chakwal vs Patoki, **E** Amino Acid biosynthesis comparison Chakwal vs Salt Range

By comparing samples via mPATH analysis, we observed that fatty acid metabolism and amino acid metabolism pathways were uniquely expressed when Patoki was compared to the Salt Range, as shown in Fig. 5A. The key difference between the two samples is the reaction that results in the formation of a certain molecule. When acetyl-CoA reacts with ACP (acyl carrier protein (ACP), the product is acetyl-ACP (acyl carrier protein), which is an intermediate in fatty acid biosynthesis. Acetyl-ACP can be used as a substrate for further fatty acid synthesis.

In contrast, when acetyl-CoA directly reacts with CO2 and ATP to form malonyl-CoA, the product is malonyl-CoA, a precursor for fatty acid biosynthesis. Malonyl-CoA is an important intermediate in fatty acid synthesis and is used to extend the length of growing fatty acid chains. Acetyl-ACP is an intermediate in fatty acid biosynthesis, whereas malonyl-CoA is the precursor. Acetyl-CoA reacts with ACP to form acetyl-ACP, which can then be used as a substrate for fatty acid biosynthesis. This pathway is more efficient if the cell needs to rapidly increase fatty acid synthesis, because it is a direct precursor for fatty acid synthesis, can be used immediately in the next step of the reaction, and is the preferred reaction in salt-range dairy cows. It was also observed that salt-range dairy cows use the most efficient pathway to synthesize palmitoyl-CoA, where hexadecanoyl-Acp directly forms palmitoyl-CoA by the enzyme acyl-ACP thioesterase, and is considered more efficient as it bypasses the need for intermediate malonyl-CoA and the enzymatic reactions involved. This pathway is energetically favorable because it bypasses the ATP-dependent carboxylation reaction required to synthesize malonyl-CoA.

In contrast, Patoki dairy cows prefer to follow pathways that convert acetyl-CoA to malonyl-CoA by reacting with CO2 and ATP, and later, malonyl-CoA reacts with ACP to form malonyl-Acp. Alternatively, acetyl-CoA directly reacts with CO2 and ATP to form malonyl-CoA, which then reacts with ACP to form malonyl-CoA. This pathway requires higher energy and expenditure of ATPs. The metabolic pathway of fatty acid biosynthesis in patoki also prefers hexadecanoyl-ACP to react with malonyl-CoA to produce palmitoyl-ACP, which is then converted to palmitoyl-CoA by acyl-ACP thioesterase and is less efficient as it requires the intermediate synthesis of malonyl-CoA and additional enzymatic reactions. There was an observed variation in the solid non-fat content of milk where Salt Range dairy cows exhibits 8.29%, and dairy cows of Patoki shows 6.34%, however, there was no significant difference in total milk fat content, which was 3.65% and 3.68% in the Salt Range and Patoki, respectively. When we compared Chakwal region dairy cows to Patoki cows shown in Fig. 5C, they also showed preference for the same pathway reactions as that of Salt Range dairy cows, where the solid non-fat content of Chakwal was 8.09% and the total fat content was 3.66%.

The milk protein content is an important quality parameter that was significantly lower in Salt Range dairy cows (2.68%) than in Patoki cows (3.43%). When Patoki dairy cows were compared to Salt Range dairy cows using mPATH analysis, as shown in Fig. 5B, we observed that Salt Range dairy cows showed preferred pathway reactions for the conversion of S-adenosyl-l-methionine (SAMe) to S-adenosyl-l-homocysteine (SAH), which is not beneficial because SAMe is a significant methyl donor that plays a critical role in protein synthesis by donating methyl groups to proteins and other molecules. This methylation process is essential for the proper folding and function of many proteins [33]. On the other hand, SAH is the product of the methylation reaction and acts as a potent inhibitor of methyltransferases, including those involved in protein synthesis. Accumulation of SAH can lead to a decrease in protein methylation and potentially impair protein synthesis without affecting milk yield. Therefore, the conversion ratio of SAMe to SAH should be maintained for the desired milk protein content. This balance is influenced by various factors, including the environment, diet, genetics, and efforts to maintain a proper ratio by supplementing diets with methyl donors, vitamins B9 and B12 to maintain a healthy methionine cycle [34], which is an active area of research in the field of nutrition and metabolism. When the Chakwal and Patoki amino acid biosynthesis pathways were compared, shown in Fig. 5D, they did not show the preferred reactions for the conversion of S-adenosyl-L-methionine (SAMe) to S-adenosyl-L-homocysteine (SAH), whereas a comparison of Chakwal to Salt Range showed that Salt Range has the preferred reactions of S-adenosyl-L-methionine (SAMe) to S-adenosyl-L-homocysteine (SAH),as shown in Fig. 5E. We further examined the abundance of such microbiota that can cause this conversion at a greater rate and found that *Bacteroides fragilis* which has enzymes called SAM hydrolases that catalyze the breakdown of SAMe to SAH, accounted for 10% of total Bacteroides in Salt Range dairy cows, 5% in Chakwal, and 3% in Patoki.

## Conclusions

To achieve sustainable production goals, it is pertinent to determine the abundance and function of microbiomes under real environmental conditions. The present study defines microbiota diversity and its functions under divergent environmental conditions and provides insight into how such microbiota can be used to improve the overall output of dairy farms.

The appropriate diets for the low performing herds can be suggested with addition of capsulated FMT by the observations of present study for dairy cows from varying environmental conditions can improve the health barriers in achieving maximum potential milk yield without compromising quality. It is of utmost importance that in changing environments and global warming, natural microbiomes are conserved for sustainable production. This study demonstrated how different gut microbiota compositions in varying environments affect milk yield and quality in dairy cows.

## Data Availability

Data is submitted to NCBI, the SRA numbers are SRR24770473, SRR24770470, SRR24770472, SRR24770471.
